# Ovarian Real-World International Consortium (ORWIC): A multicentre, real-world analysis of epithelial ovarian cancer treatment and outcomes

**DOI:** 10.3389/fonc.2023.1114435

**Published:** 2023-01-27

**Authors:** Sue Cheeseman, Bethany Levick, Will Sopwith, Hayley Fenton, Eun Ji Nam, DongKyu Kim, Subin Lim, Elodie Martin, Jean-Sébastien Frenel, François Bocquet, Paul Kubelac, Patriciu Achimas-Cadariu, Catalin Vlad, Marion Chevrier, Roman Rouzier, Matthieu Carton, Joana Savva-Bordalo, Marta Magalhães, Marina Borges, Andrea Wolf, Sven Becker, Nicolas Niklas, Mariana Guergova-Kuras, Geoff Hall

**Affiliations:** ^1^ Leeds Cancer Center, Leeds Teaching Hospitals NHS Trust, Leeds, United Kingdom; ^2^ Oncology Evidence Network, IQVIA, London, United Kingdom; ^3^ Department of Obstetrics and Gynecology, Institute of Women’s Medical Life Science, Yonsei Cancer Center, Severance Hospital, Yonsei University College of Medicine, Seoul, Republic of Korea; ^4^ Department of Family Medicine, Yonsei University College of Medicine, Seoul, Republic of Korea; ^5^ Real-World Evidence Team, ALYND, Yonsei University Health System, Seoul, Republic of Korea; ^6^ Department of Biostatistics, Clinical Trial Sponsor Unit, Institut de Cancérologie de l’Ouest, Nantes-Angers, France; ^7^ Oncology Department, Institut de Cancérologie de l’Ouest, Center for Research in Cancerology and Immunology, INSERM UMR 1232, Nantes University and Angers University, Nantes-Angers, France; ^8^ Data Factory and Analytics Department, Institut de Cancérologie de l’Ouest, Law and Social Change Laboratory, Faculty of Law and Political Sciences, CNRS UMR 6297, Nantes University, Nantes-Angers, France; ^9^ The Oncology Institute “Prof. Dr. Ion Chiricuta”, Iuliu Hatieganu University of Medicine and Pharmacy, Cluj Napoca, Romania; ^10^ Department of Biostatistics, Institut Curie, Paris Sciences et Lettres (PSL) University, Paris, France; ^11^ Department of Breast and Gynecological Surgery, Institut Curie, Paris, France; ^12^ Department of Medical Oncology, Portuguese Oncology Institute of Porto (IPO-Porto) Porto, Porto, Portugal; ^13^ Cancer Epidemiology Group-Research Center, IPO Porto, Comprehensive Cancer Center (Porto.CCC), RISE@CI-IPOP (Health Research Network), Porto, Portugal; ^14^ Management, Outcomes Research and Economics in Healthcare Group-Research Center, IPO Porto, Comprehensive Cancer Center (Porto.CCC), RISE@CI-IPOP (Health Research Network), Porto, Portugal; ^15^ Internal Medicine, Universitätsklinikum Frankfurt am Main, Frankfurt, Germany; ^16^ Oncology Evidence Network, IQVIA Commercial GmbH and Co. OHG, Frankfurt am Main, Frankfurt, Germany; ^17^ Oncology Evidence Network, IQVIA, La Défense, France

**Keywords:** epithelial ovarian cancer, chemotherapy, debulking surgery, real-world, treatment, Europe

## Abstract

**Introduction:**

Much drug development and published analysis for epithelial ovarian cancer (EOC) focuses on early-line treatment. Full sequences of treatment from diagnosis to death and the impact of later lines of therapy are rarely studied. We describe the establishment of an international network of cancer centers configured to compare real-world treatment pathways in UK, Portugal, Germany, South Korea, France and Romania (the Ovarian Real-World International Consortium; ORWIC).

**Methods:**

3344 patients diagnosed with EOC (2012-2018) were analysed using a common data model and hub and spoke programming approach applied to existing electronic medical records. Consistent definition of line of therapy between sites and an efficient approach to analysis within the limitations of local information governance was achieved.

**Results:**

Median age of participants was 53-67 years old and 5-29% were ECOG >1. Between 62% and 84% of patients were diagnosed with late-stage disease (FIGO III-IV). Sites treating younger and fitter patients had higher rates of debulking surgery for those diagnosed at late stage than sites with older, more frail patients. At least 21% of patients treated with systemic anti-cancer therapy (SACT) had recurrent disease following second-line therapy (2L); up to 11 lines of SACT treatment were recorded for some patients. Platinum-based SACT was consistently used across sites at 1L, but choices at 2L varied, with hormone therapies commonly used in the UK and Portugal. The use (and type) of maintenance therapy following 1L also varied. Beyond 2L, there was little consensus between sites on treatment choice: trial compounds and unspecified combinations of other agents were common.

**Discussion:**

Specific treatment sequences are reported up to 4L and the establishment of this network facilitates future analysis of comparative outcomes per line of treatment with the aim of optimizing available options for patients with recurrent EOC. In particular, this real-world network can be used to assess the growing use of PARP inhibitors. The real-world optimization of advanced line treatment will be especially important for patients not usually eligible for involvement with clinical trials. The resources to enable this analysis to be implemented elsewhere are supplied and the network will seek to grow in coverage of further sites.

## 1 Introduction

Epithelial ovarian cancer (EOC) (including primary peritoneal cancer and fallopian tube cancer), is a gynecological malignancy resulting in at least 4,000 deaths in the UK and 140,000 deaths world-wide each year (1, 2). EOC is a heterogeneous disease, comprising several morphological or histological types with distinct epidemiologic, molecular, and clinical features. Surveillance data suggests that survival outcomes differ between these morphologies (3) and the distribution of morphologies and experience of treatment differs internationally (4, 5).

The optimal management of EOC is surgical cytoreduction (debulking) followed by chemotherapy. The outcome of surgery (i.e. volume of residual disease) remains one of the most important predictors of survival, and neoadjuvant chemotherapy may be considered where the likelihood of optimal debulking is in doubt or is associated with unacceptable morbidity (6). For many years, primary treatment regimens have been a combination of platinum-based chemotherapy and taxane (such as paclitaxel) (6). Bevacizumab (a monoclonal antibody binding vascular endothelial growth factor) has demonstrated some modest improvement on PFS when given in combination with intravenous chemotherapy and as a maintenance therapy and gains in overall survival in high-risk stage III-IV patients with residual disease and ascites (7, 8). Nevertheless, bevacizumab was authorized (in 2011) by the European Medicines Agency as a first and second line treatment (in combination with chemotherapy) (9), with subsequent studies showing improved outcomes in platinum-sensitive (10–12) and platinum-resistant disease (13). Bevacizumab is now considered standard of care in many countries (14). More recently, patients with platinum-sensitive, relapsed ovarian cancer have been shown to derive significant benefit from the addition of a Poly-ADP-ribose polymerase (PARP) inhibitor as maintenance, especially (but not exclusively) with a germline or somatic mutation in BRCA1 or BRCA2 (15, 16). Several studies have also shown benefit of PARPi given as monotherapy for patients with multiple previous lines of treatment (17), but innate mechanisms of resistance (18) mean that effective combination therapies are also needed. Studies assessing the benefit of immune checkpoint inhibitors for recurrent disease are ongoing (19, 20).

Despite excellent responses to chemotherapy and surgery, only a small minority of patients achieve long-term cure whilst most patients experience relapse of their disease months or even years later and require further treatment (21). In addition to tumor biology/histology, number of prior lines of treatment and prior response, treatment of relapsed disease is determined by the time since the last platinum-based chemotherapy or the ‘platinum-free interval’ (PFI); patients relapsing at least six months since last platinum-based treatment are considered ‘platinum-sensitive’ and those relapsing earlier, ‘platinum- resistant’ (22). Given the advances in detection of relapsed disease and the emergence of new targeted therapies, some authors argue that these traditional definitions are becoming a less reliable indication for treatment choices (17) and treatment for relapsed disease may become increasingly individualized. In 2015, a more generic algorithm was introduced to replace PFI: therapy-free interval (TFI) (23).

The impact of surgery for recurrent disease (secondary surgical cytoreduction) is uncertain, with recent trials apparently showing conflicting results (24, 25). Any benefit on overall survival (OS) depends upon complete resection (at secondary surgical cytoreduction), and likelihood of this outcome appears to be independently associated with individual patient performance score (PS), residual disease following *initial* debulking surgery and cancer stage at original diagnosis (26).

Clinical trials in EOC most often examine therapy at a particular phase of treatment, for example at first line following diagnosis or second line following first recurrence. Few, if any, major studies have examined the management of patients from diagnosis to death, reporting the sequence of treatment used and the impact and benefit achieved by each line of therapy. International comparison of treatment outside the context of clinical trials is also rare, particularly in the era of targeted therapy with PARP inhibitors, monoclonal antibodies and immune checkpoint inhibitors. There is a current unmet need to optimize treatment for recurrent EOC (27).

The present study aims to describe the characteristics of patients with EOC in a real-world setting treated in 7 cancer centers in 6 different countries across Europe and Asia, including their complete treatment pathways from diagnosis to last observation.

## 2 Materials and methods

### 2.1 Study design

The study is a retrospective observational cohort study, using electronic health record (EHR) data collected as part of standard patient care in participating treatment centers. Available data was a mix of structured data items and unstructured text recorded in clinical notes. Where necessary, structured data was enriched at each study site through manual curation of clinical notes by local clinical teams. The study uses a “hub and spoke” analytical approach, with participating sites locally executing shared analytical scripts that were centrally coded to a common data model (CDM). This approach followed established design principles. preventing the need for patient-level data to leave the sites, so allowing the study to be conducted across sites working under diverse data protection legislation (28). The study was locally approved by the ethics committee at each treatment center, in accordance with the Declaration of Helsinki guidelines for biomedical research.

### 2.2 Study population

Study sites were Leeds Teaching Hospitals Trust (‘LTHT’, Leeds, UK), Instituto Português de Oncologia Porto (‘IPO’, Porto, Portugal), Yonsei University Health System (‘Yonsei’, Seoul, South Korea), University Hospital Frankfurt am Main (‘Frankfurt’, Frankfurt am Main, Germany), Institut de Cancérologie Ouest (‘ICO’, Saint-Herblain and Angers, France), Institut Curie (‘Curie’, Paris, France) and Institute of Oncology Prof Dr. Ion Chiricuta (‘Cluj’, Cluj-Napoca, Romania).

The study cohort was adult patients (at least 18 years old) with a first diagnosis of EOC (including all primary peritoneal carcinoma and fallopian tube cancer) or carcinosarcoma (mixed Mullerian tumor) between 1st January 2012 and 31st December 2018. Patients with sarcomas, stromal tumors and germ cell tumors or a clinically significant second primary malignancy (other than breast cancer and non-melanoma skin cancer) before or after the diagnosis of EOC were excluded (see Data Guide – **supplementary information**). Other exclusions were patients with a non-continuous care record following diagnosis (due to an episode of treatment being undertaken elsewhere and data not being available to the study site investigators) or patients opting out of local data sharing agreements or secondary research participation.

### 2.3 Study variables

The study period was from 1st January 2012 to 31st December 2019. Patient data was collected from the date of primary diagnosis of EOC (index date) until 31st December 2019, end of available data or death.

**Figure 1:**
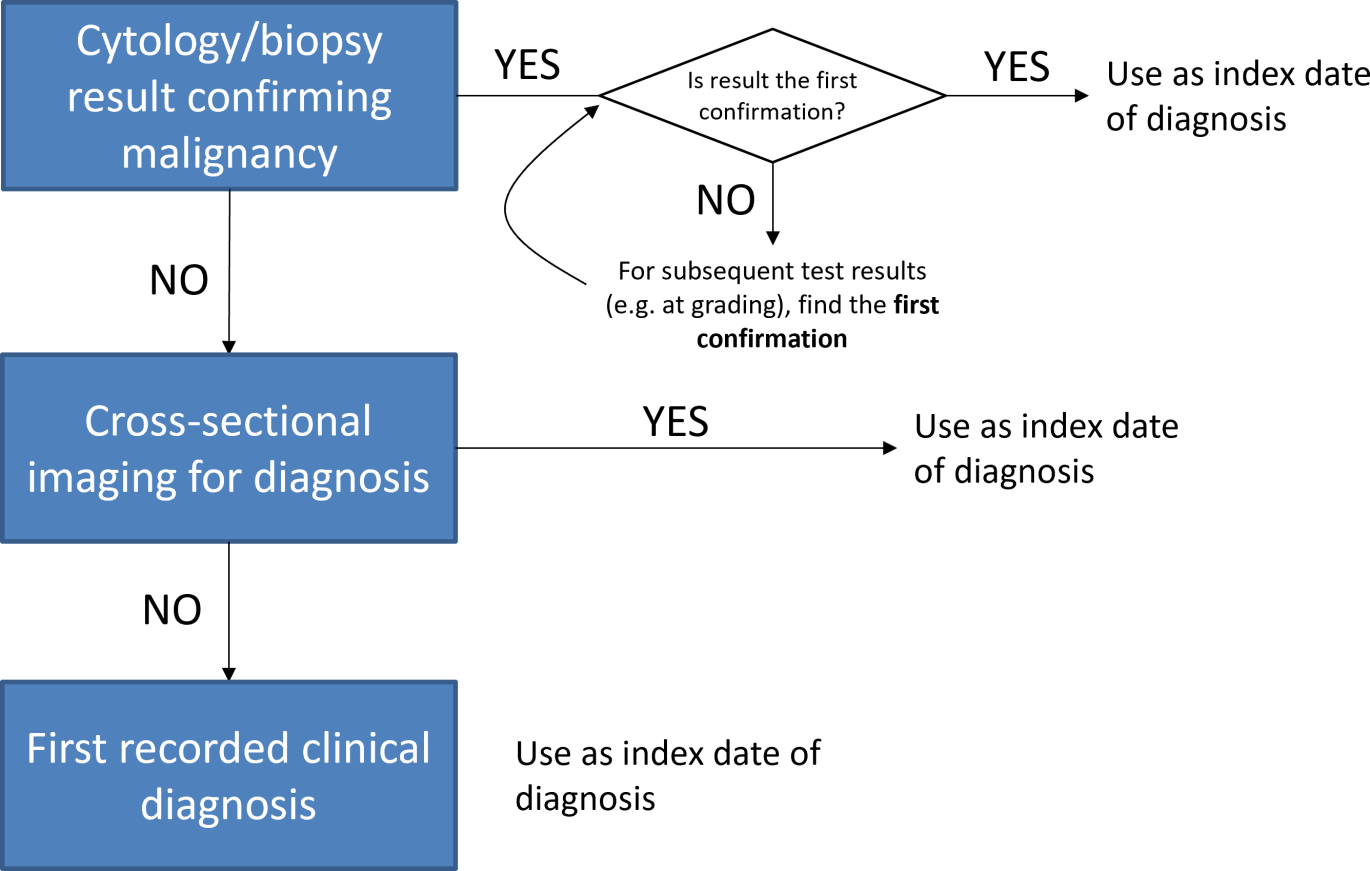
Hierarchy used to define index date of diagnosis in real-world data across study sites.

Index date was defined through an iterative consensus building process (see Results) between study site principal investigators. The final study definition was the first date of histological confirmation of malignancy by biopsy or cytology. Where histology or cytology was not done, the first date of cross-sectional imaging or first clinical diagnosis was used (**Figure 1**).

The study data guide detailing the CDM is available in **supplementary data** and the compilation of a data dictionary described in results. In summary, patient characteristics, tumor characteristics and treatment events (surgery, systematic anti-cancer therapy (SACT), radiotherapy) were extracted from each site’s EHR. Cancer morphology was defined as high-grade serous, low grade serous, serous with no grade defined, endometrioid, clear cell, mucinous, carcinosarcoma, mixed, undifferentiated, small cell carcinoma, missing, unknown or other. In general, the CDM maps to international standards where available (e.g. ICD10, ICD-O-3). Otherwise, definitions were agreed through a similar review by study clinicians and statistical programmers.

### 2.4 Study analysis

Characterization of the patient cohort and sub-groups, and the therapy they received following diagnosis with EOC was through descriptive analyzes as follows:

- Clinical characteristics of the population (age, Eastern Cooperative Oncology Group (ECOG) score (29), BRCA variant status) and of their cancer (site, FIGO stage (30), morphology, grade)
- The frequency of debulking surgery and SACT use, and the number of lines of therapy (LoTs) of SACT administered
- The distribution of therapeutic and maintenance agents used at each LoT of SACT
- The time to next treatment (TTNT) from diagnosis to the second LoT - TTNT was used as a real-world proxy for progression following first LoT treatment
- Overall survival (OS) since diagnosis

Site level analysis was conducted according to consistent principles agreed between study sites. Site level results were summarized as aggregated tables or figures. Categorical variables were summarized as counts and percentages, and continuous variables as means, medians, interquartile ranges (IQR) and standard deviations (SD). Median time to event (TTNT and OS) was calculated using the Kaplan Meier product-limit method.

### 2.5 Data management and statistical programming

Each study site generated an analysis dataset from their EHR using the agreed CDM. Data were analyzed locally using a distributed R package (‘openovary’), code that had been developed centrally at one study site (LTHT) [R version 3.6.3 (31)]. Aggregated outputs were disseminated amongst the network PIs for review. All patient level data was retained within local hospital systems.

## 3 Results

### 3.1 Developing a common data model

#### 3.1.1 Common derivation of index date and line of therapy

All data extraction and management were done locally at each study site. To ensure consistency of definition, the lead PI developed 3 realistic but fictitious patient treatment pathways. Pathways included surgery, SACT (including maintenance therapy) and radiography events. From these scenarios, each PI was asked to parse the described courses of treatment into distinct programs of related events and identify the most appropriate index date for the definition of outcome. Each site’s assessment was independently recorded with the lead PI (Leeds) and a consolidated summary of views was prepared. Areas of difference in perspective on treatment program and index date were discussed on a research call between PIs until consensus of study definitions was reached.

The consensus reached on appropriate classification of index date is shown (**Figure 1**). Regarding treatment program, there was baseline agreement between sites on the grouping of real-world events into distinct treatment programs. However, grouping of specific drug regimens into distinct programs was more diverse between sites, and sometimes varied between practitioners within individual treatment centers. From the consensus reached, distinct LoTs and maintenance were defined before data extraction and analysis.

Regarding surgery, perspectives differed between sites on the definition and recording of surgical events in EHRs. Areas of particular focus included the distinction within existing data systems between significant biopsy and cytoreduction, the coding of patients for whom surgery was initiated but a full debulking procedure was not possible and the grading of residual disease. Some study sites had extensive surgical procedure metrics (for example, time in theatre, associated complications) that were mapped in the final CDM but could not be collected from every site. From the consensus reached through PI discussion, a data dictionary and CDM were developed.

#### 3.1.2 Compilation of data dictionary and hub and spoke analytical code approach

Legislation governing the protection of personal and healthcare data differs between the host nations of the included study sites. Centrally coded scripts were produced to allow distributed analysis, in preference to the time cost of establishing multiple data sharing agreements. Analytical aims and outputs were first agreed between the clinical and data teams before constructing the necessary CDM. The definition of final output tables, including the most appropriate groupings and stratifications of the study cohort, was agreed iteratively over sequential data releases.

**Figure 2:**
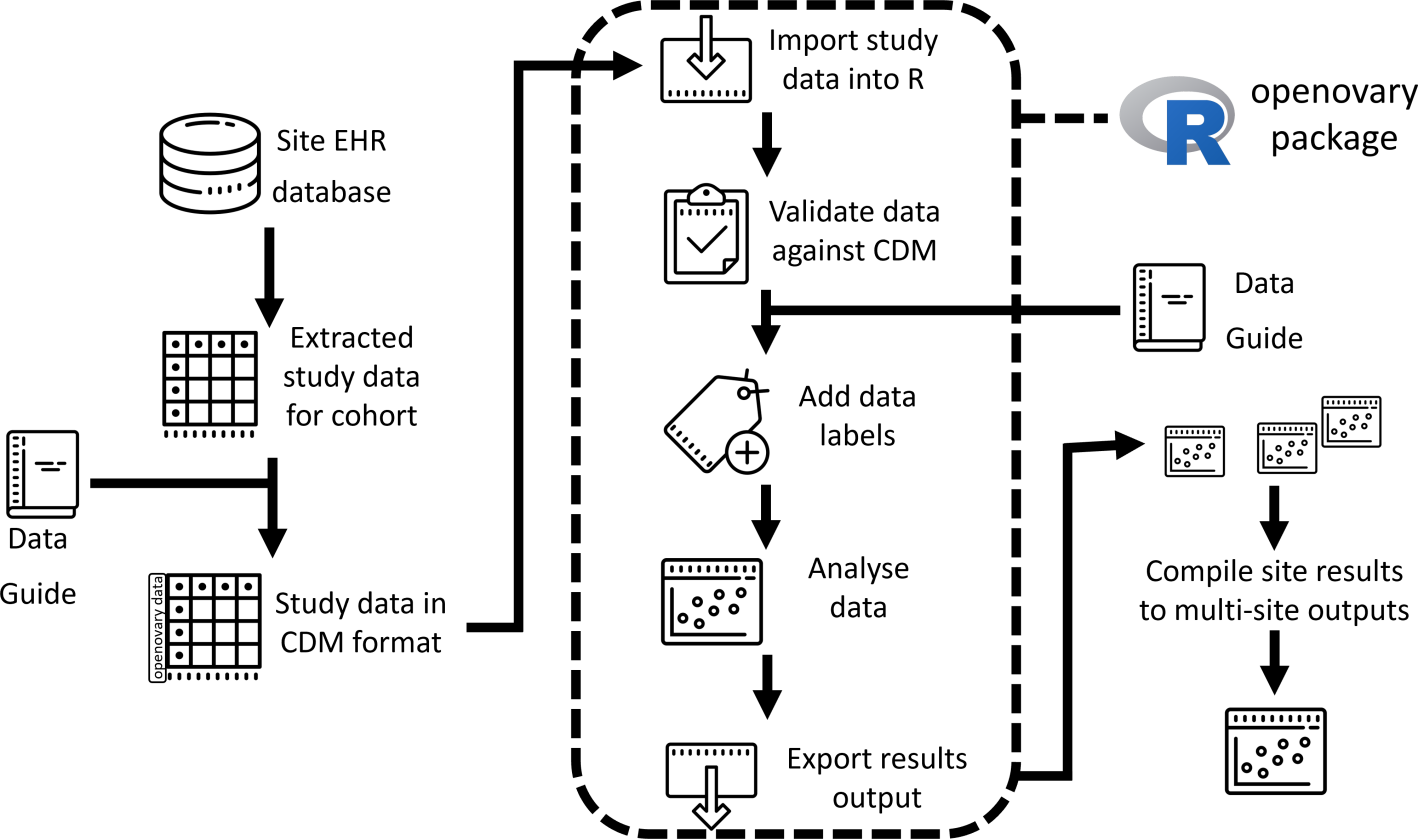
Summary schematic of the data access and analytical process at each study site.

Sites were provided with a data guide (see **supplementary data**) detailing variable-level derivations and validation rules, general data coding principles, and conversions/codes from international coding systems. A bespoke package of analysis scripts was programmed centrally (at LTHT), designed to validate each site’s data against the CDM and generate standard outputs of tables and time to event analyzes. The package (‘openovary’ available in **supplementary data**) includes functions, numerical summaries and Kaplan-Meier analysis of overall survival. Each study site translated their local data to the study CDM before analysis using the openovary package (**Figure 2**). The resulting analytical output was presented for each site.

### 3.2 Patient characteristics

The total study cohort identified across the research network numbered 3344 patients diagnosed with EOC between 1st January 2012 and 31st December 2018. The median age of patients diagnosed with EOC ranged from 53 to 67 years old across the study sites (**Table 1**). Patients diagnosed at Cluj (Romania) and Yonsei (South Korea) were, in general, younger with 65.1% and 74.5% (respectively) first diagnosed before the age of 60 years old. The oldest cohort was described in LTHT (UK), where 26.2% of patients were diagnosed at 75 years old or older. Distribution of ECOG status varied considerably across sites, and cohorts with higher median age tended to have a higher proportion of patients with ECOG of 2 or higher. Advanced stage disease (FIGO III or IV) was also more commonly diagnosed at sites with higher median age.

**Table 1:** Characteristics of ovarian cancer patients treated at 7 participating study sites between 2012 and 2018.

		Cluj		Curie		Frankfurt		ICO		IPO		LTHT*		Yonsei	
	Cohort	446		428		140		696		268		515		851	
Age (years)	Median	55		63		59		65		66		67		53	
	Q1-Q3	46-63		52-71		51-68		56-72		57-74		58-75		46-61	
Age Group (years)	<45	81	18.2%	46	10.7%	17	12.1%	45	6.5%	12	4.5%	20	3.9%	166	19.5%
	45-59	209	46.9%	127	29.7%	54	38.6%	174	25.0%	75	28.0%	125	24.3%	440	51.7%
	60-74	139	31.2%	193	45.1%	51	36.4%	329	47.3%	114	42.5%	235	45.6%	217	25.5%
	75+	17	3.8%	62	14.5%	18	12.9%	148	21.3%	67	25.0%	135	26.2%	28	3.3%
ECOG	0 - Normal	197	49.7%	60	46.9%	26	46.4%	202	38.2%	109	50.9%	143	35.1%	211	74.8%
	1 - Light Work	179	45.2%	48	37.5%	23	41.1%	243	45.9%	49	22.9%	147	36.1%	56	19.9%
	2 - Ambulatory >50%	16	4.0%	14	10.9%	4	7.1%	66	12.5%	29	13.6%	69	17.0%	14	5.0%
	3 - Ambulatory <50%	4	1.0%	6	4.7%	3	5.4%	13	2.5%	13	6.1%	<50		1	0.4%
	4 - Continuous Care	0		0		0		5	0.9%	14	6.5%	<6		0	
	Not available	50	NA	300	NA	84	NA	167	NA	54	NA	108	NA	569	NA
Primary tumor site	Ovary (C56)	423	94.8%	326	76.2%	133	95.0%	682	98.0%	227	84.7%	307	59.6%	790	92.8%
	Fallopian tube (C57)	22	4.9%	41	9.6%	6	4.3%	4	0.6%	40	14.9%	38	7.4%	23	2.7%
	Primary peritoneal: mullerian (C48)	1	0.2%	61	14.3%	1	0.7%	10	1.4%	1	0.4%	170	33.0%	38	4.5%
	Missing/Not known	0	NA	0	NA	0	NA	0	NA	0	NA	0	NA	0	NA
Tumor FIGO stage	I	101	22.6%	35	11.0%	21	15.4%	73	12.5%	39	15.2%	56	10.9%	232	29.4%
	II	37	8.3%	18	5.7%	8	5.9%	27	4.6%	18	7.0%	<30		68	8.6%
	III	259	58.1%	149	46.9%	72	52.9%	323	55.2%	106	41.2%	293	57.0%	279	35.3%
	IV	49	11.0%	116	36.5%	35	25.7%	162	27.7%	94	36.6%	140	27.2%	211	26.7%
	Missing/Unknown	0	NA	110	NA	4	NA	111	NA	11	NA	<6	NA	61	NA
Tumor morphology	High grade serous	288	64.6%	243	56.8%	87	62.1%	472	67.8%	103	38.4%	350	68.0%	442	51.9%
	Endometrioid	32	7.2%	12	2.8%	12	8.6%	22	3.2%	15	5.6%	43	8.3%	97	11.4%
	Clear Cell	10	2.2%	19	4.4%	4	2.9%	28	4.0%	20	7.5%	9	1.7%	99	11.6%
	Mucinous	28	6.3%	17	4.0%	7	5.0%	14	2.0%	13	4.9%	15	2.9%	68	8.0%
	Low grade serous	43	9.6%	70	16.4%	7	5.0%	18	2.6%	13	4.9%	25	4.9%	24	2.8%
	Serous, unknown grade	6	1.3%	0		9	6.4%	3	0.4%	31	11.6%	20	3.9%	24	2.8%
	Mixed	23	5.2%	15	3.5%	0		12	1.7%	10	3.7%	8	1.6%	13	1.5%
	Carcinosarcoma	1	0.2%	8	1.9%	0		9	1.3%	11	4.1%	36	7.0%	15	1.8%
	Other/Undifferentiated/NK	12	2.7%	42	9.8%	14	10.0%	117	16.8%	51	19.0%	<6		50	5.9%
	Small Cell	3	0.7%	2	0.5%	0		1	0.1%	1	0.4%	<6		19	2.2%
Tumor grade	Well differentiated	77	18.6%	18	5.9%	13	10.5%	42	8.4%	27	16.7%	34	7.4%	82	9.6%
	Moderately differentiated	54	13.1%	26	8.6%	24	19.4%	38	7.6%	12	7.4%	13	2.8%	153	18.0%
	Poorly differentiated	282	68.3%	259	85.5%	87	70.2%	426	85.0%	123	75.9%	414	89.8%	428	50.3%
	Missing/Invalid	33	NA	125	NA	16	NA	190	NA	106	NA	54	NA	188	NA

*small numbers masked for this center; NA, not applicable.

Within the definition of EOC, the distribution of specific tumor sites was markedly different for some study sites. Although malignancy of the ovary (ICD-10 C56) predominated in the research network, primary peritoneal cancer (C48) was commonly the diagnosis recorded in LTHT (UK) and Curie (France). The most common morphology of EOC tumor at all study sites was high grade serous. The proportions of study cohort where data was missing or unknown within site EHRs also varied across sites and by variable (**Table 1**).

The proportion of patients tested for BRCA at EOC diagnosis ranged from 16.1% (72/446; Cluj, Romania) to 68.9% (295/428; Curie, France), indicating different testing practices across the network (**Table 2**). Between 2.3% and 33.3% of patients tested for BRCA were found to possess a pathogenic variant, which was most commonly germline BRCA1.

**Table 2:** BRCA testing per site and proportion of pathogenic variants (‘variant’) identified.

	Cluj (n=446)			Curie (n=428)			ICO (n=696)			IPO (n=268)			LTHT (n=515)			Yonsei (n=851)		
	Tests	Variant	% variant	Tests	Variant	% variant	Tests	Variant	% variant	Tests	Variant	% variant	Tests	Variant	% variant	Tests	Variant	% variant
Germline BRCA1	72	24	33.3%	295	41	13.9%	356	52	14.6%	131	7	5.3%	230	24	10.4%	444	69	15.5%
Germline BRCA2	72	4	5.6%	282	23	8.2%	344	17	4.9%	131	7	5.3%	217	19	8.8%	447	52	11.6%
Year routine testing started	2016			2009			2008			2015			2016			2002		
Somatic BRCA1	8	–		99	15	15.2%	48	4	8.3%	131	3	2.3%	*	<6		175	12	6.9%
Somatic BRCA2	8	–		94	7	7.4%	46	2	4.3%	131	3	2.3%	*	<6		175	8	4.6%
Year routine testing started	2016			2014			2014			2015			2015			2017		

Data not available in Frankfurt. *Number of somatic tests could not be reliably distinguished from germline testing in LTHT. BRCA testing may have been performed at diagnosis or at relapse events and these time points are not distinguished in this data.

### 3.3 Surgical outcome

**Figure 3:**
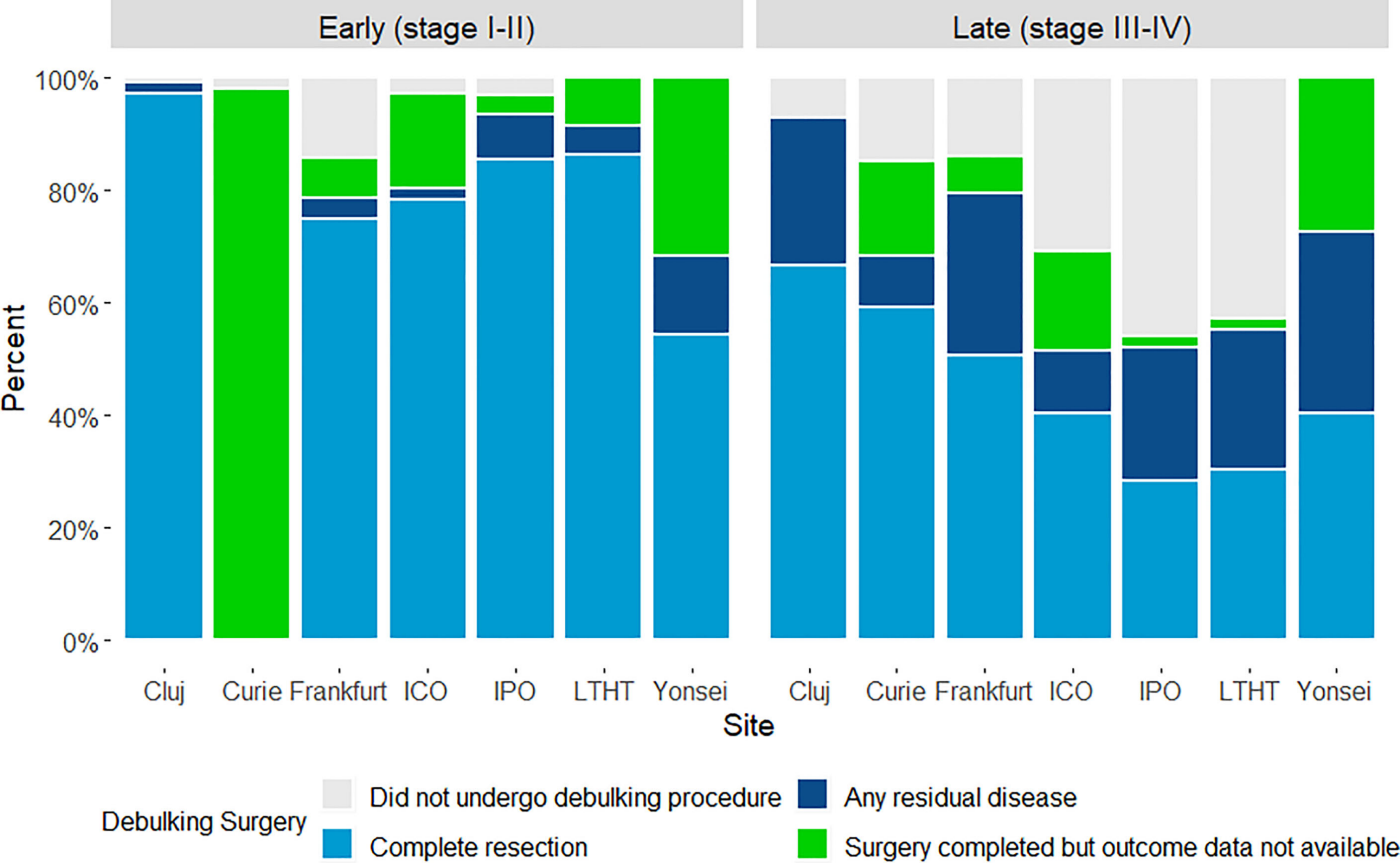
Outcome achieved following primary surgical debulking surgery, by study site and FIGO disease stage.

Where early-stage disease (FIGO I-II) was diagnosed, at least 75% of patients had complete resection verified as an outcome following debulking surgery (**Figure 3**) (in all but two study sites; outcome of surgery was recorded less frequently for patients at Yonsei and not available for Curie). Where late-stage disease (FIGO III-IV) was diagnosed, debulking surgery was less frequently recorded and residual disease post-surgery was more common. Patients at IPO and LTHT were the least likely to undergo debulking surgery at late stage (**Figure 3**). Across all sites, late-stage patients undergoing debulking surgery appeared to be younger, less likely to have stage IV disease and more likely to have an ECOG score of less than 2, when compared with late-stage patients not undergoing debulking surgery (**Table 3**).

**Table 3:** Characteristics of patients with late-stage disease at diagnosis, by receipt of debulking surgery.

	Cluj				Curie				Frankfurt				ICO				IPO				LTHT			
	no debulking		debulking		no debulking		debulking		no debulking		debulking		no debulking		debulking		no debulking		debulking		no debulking		debulking	
Late-stage cohort	22	100%	286	100%	39	100%	226	100%	14	100%	93	100%	139	100%	346	100%	97	100%	103	100%	186	100%	247	100%
Median age (Q1-Q3)	64 (56-69)		55 (48-63)		73 (67-79.5)		63 (53-70)		67 (58-79)		59 (52-68)		70 (63-78)		65 (57-71)		72 (61-77)		63 (55-71)		74 (65-79)		65 (56-72)	
Stage IV	8	36.4%	41	14.3%	29	74.4%	87	38.5%	7	50.0%	28	30.1%	83	59.7%	79	22.8%	68	70.1%	26	25.2%	85	45.7%	55	22.3%
ECOG 0-1	17	94.4%	238	99.2%	13	72.2%	68	88.3%	0	NA	33	86.8%	76	65.5%	261	89.4%	31	42.5%	77	88.5%	68	47.9%	159	80.3%
ECOG 2-4	1	5.6%	2	0.8%	5	27.8%	9	11.7%	1	NA	5	13.2%	40	34.5%	31	10.6%	42	57.5%	10	11.5%	74	52.1%	39	19.7%
ECOG NK	4	NA	46	NA	21	NA	149	NA	13	NA	55	NA	23	NA	54	NA	24	NA	16	NA	44	NA	49	NA
Primary debulking			187	65.4%			53	23.5%			84	90.3%			122	35.6%			62	60.2%			66	27.2%
Interval debulking			27	9.4%			154	68.1%			8	8.6%			127	37.0%			21	20.4%			167	68.7%
Delayed debulking			72	25.2%			19	8.4%			1	1.1%			94	27.4%			20	19.4%			<15	NA
Missing/Unknown			0	NA			0	NA			0	NA			3	NA			0	NA			<6	NA

Yonsei not shown as all late-stage patients received debulking surgery. NA, not applicable.

### 3.4 Treatment with SACT

**Figure 4:**
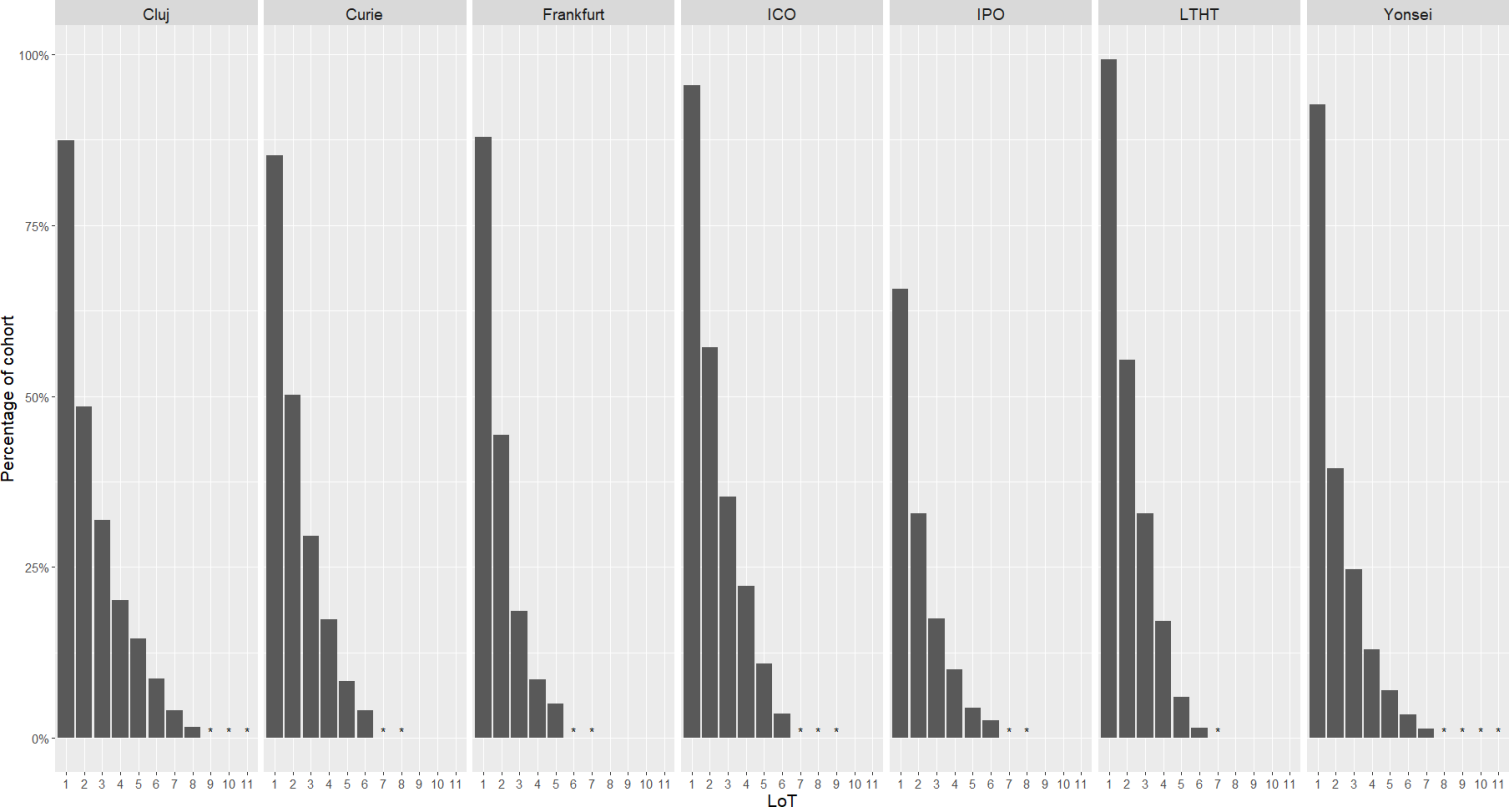
Proportion of cohort receiving systemic anti-cancer therapy (SACT) following diagnosis, including treatment attrition through successive lines of therapy (LoT), by study site. *indicates where cohort treated was <6 patients.

The proportion of patients treated with SACT following diagnosis ranged from 65.7% (176/268; IPO) to 99.2% (511/515; LTHT), with all sites except IPO treating at least 87% (**Figure 4**). Of those treated with SACT, at least 50% went on to receive a second LoT in all sites except Yonsei, where the proportion was 42.6%. Proportions of SACT-treated patients receiving 3L treatment ranged from 21.1% (26/123; Frankfurt) to 37.0% (246/664; ICO) and the maximum number of LoT recorded for a patient in the study cohort was 11 (Cluj and Yonsei). Patients were least likely to be treated beyond 3L in Frankfurt.

**Figure 5:**
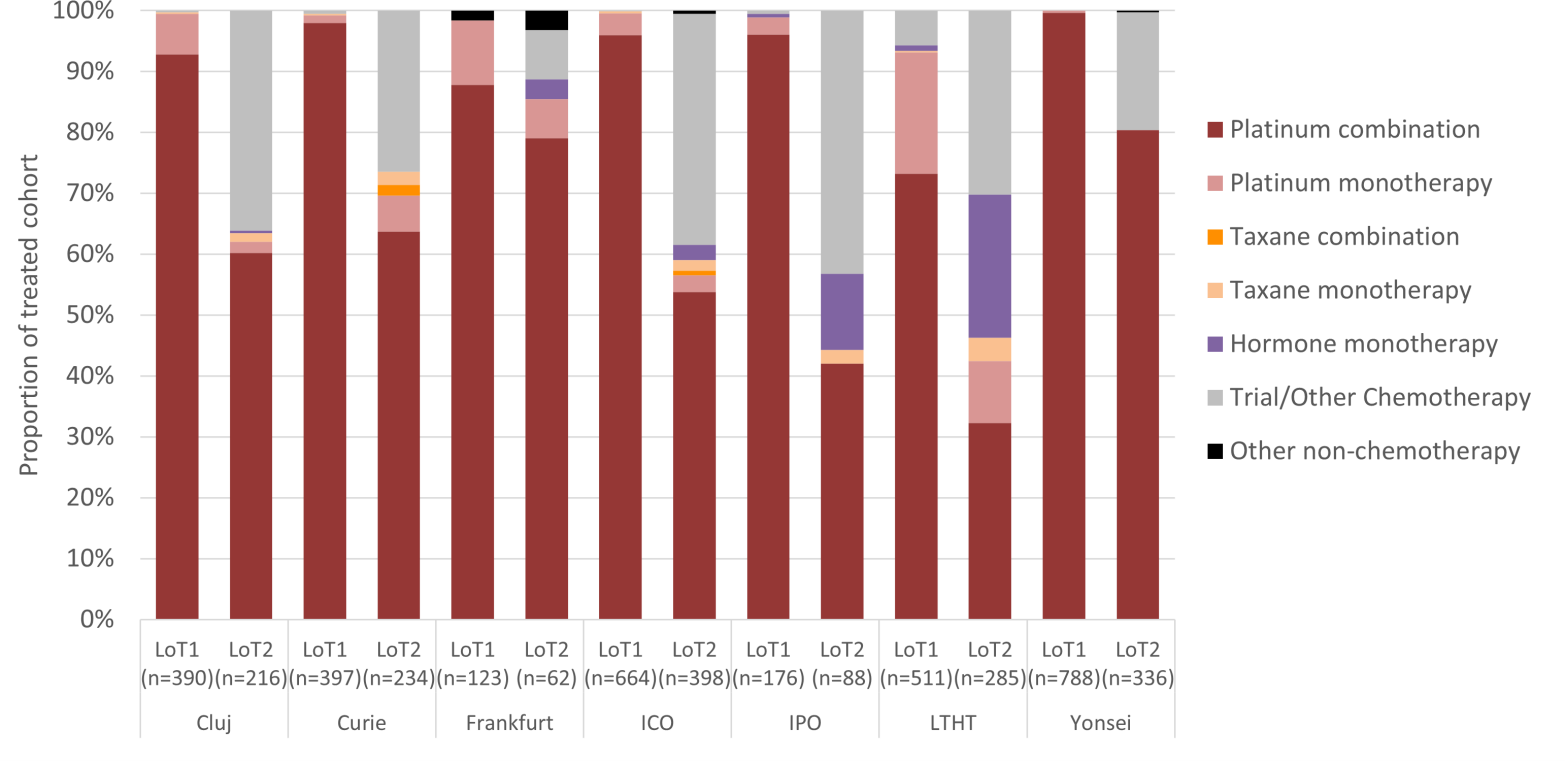
Proportion of SACT class received at first and second LoT, by study site.

Platinum chemotherapy was used as 1L treatment almost exclusively across sites, with the exception of LTHT, where 5.7% (29/511) of patients received a trial drug or other chemotherapy (**Figure 5**). Platinum was most frequently used in combination with another therapy (usually a taxane) and use as monotherapy varied across sites, with patients diagnosed at LTHT most frequently receiving this at 1L (20.0% of patients, 102/511). Treatment classes used at 2L were more diverse than at 1L, with platinum use across sites ranging from 42.0% (37/88; IPO) to 85.5% (53/62; Frankfurt). The proportion of patients receiving a trial drug or ‘other’ chemotherapy at 2L was notable across all sites (between 19.3% (65/336; Yonsei) and 43.2% (38/88; IPO)), but regular use of hormone monotherapy was more restricted to LTHT and IPO. All sites except Yonsei and Frankfurt included some patients treated with a taxane at 2L, sometimes in combination with another agent.

**Figure 6:**
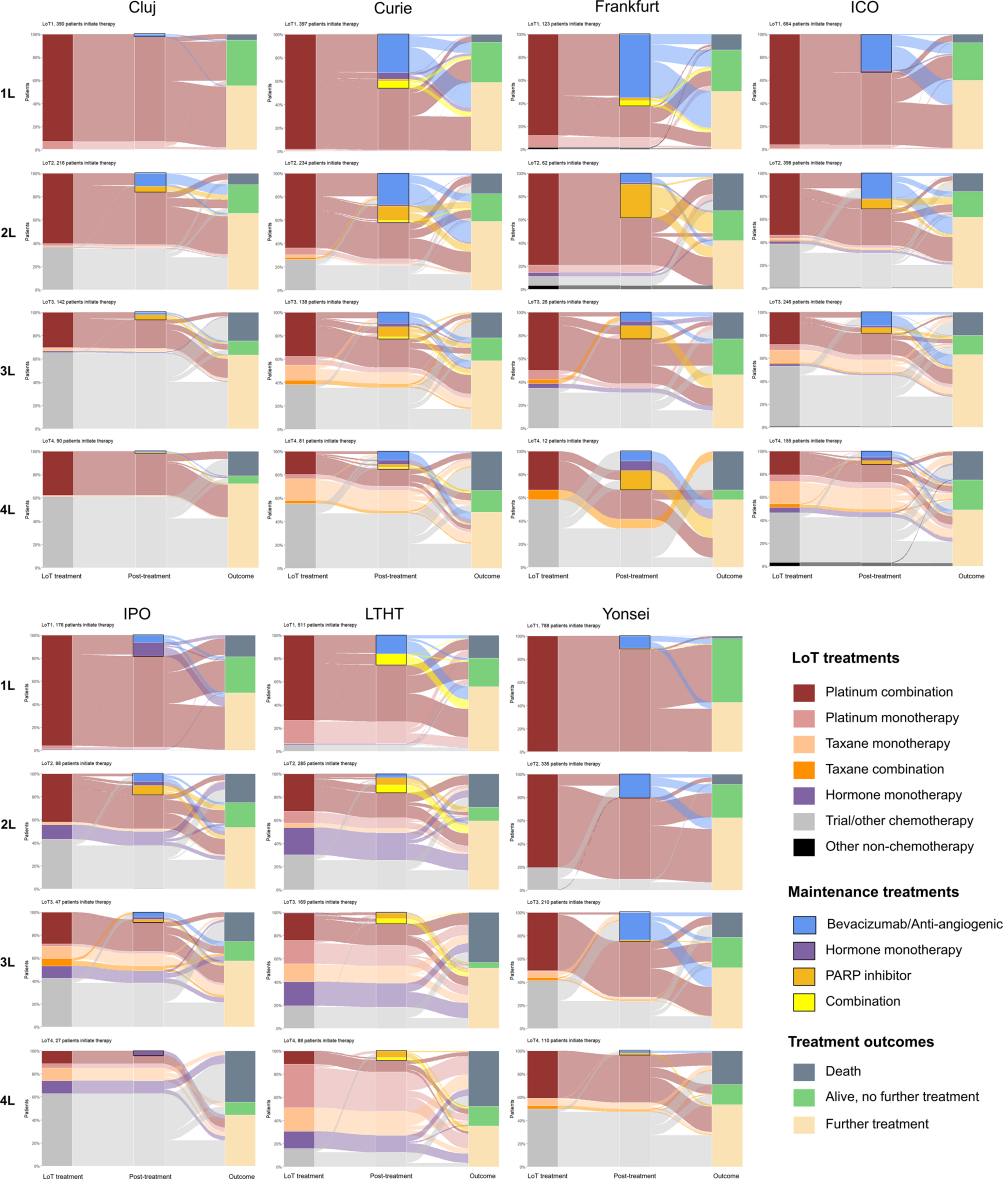
Treatment pathways and outcomes for the first 4 lines of SACT treatment (1L to 4L), by study site. Class of treatment regimen is shown in the left-hand column (‘LoT treatment’), subsequent maintenance therapy (where used) is indicated in the middle column (‘post treatment’) by a solid outline and treatment outcome is in the right-hand column (‘outcome’).

Following 1L treatment, the proportion of patients receiving maintenance therapy varied between 2.3% (9/390; Cluj) and 62.6% (77/123; Frankfurt) and bevacizumab was the most common single therapy used (**Figure 6**). Alternative maintenance therapies of note following 1L were hormone monotherapy (IPO) and PARPi (Frankfurt) and combined maintenance therapy (usually bevacizumab combined with hormone or with PARPi) was frequently used at LTHT. Overall, between 42.6% (336/788; Yonsei) and 60.0% (398/664; ICO) of patients treated at 1L remained alive and received further lines of treatment. Patients treated with maintenance therapy appeared to be more likely to receive further SACT treatment than those who were not (range between 50.0% (44/88; Yonsei) and 75.0% (99/132; LTHT)). Death following 1L treatment was most frequently reported as outcome for patients diagnosed at LTHT (19.8%, 101/511) and IPO (18.8%, 33/176) (**Figure 6**).

In addition to the distribution of maintenance therapies described after 1L treatment (above), PARPi was more commonly included as maintenance therapy after 2L, particularly for patients diagnosed at Frankfurt (**Figure 6**). PARPi was exclusively used as maintenance following platinum treatment (or trial compounds including platinum). In most sites, bevacizumab (including bevacizumab combinations) remained more commonly used as maintenance following 2L than PARPi, except for Frankfurt (where PARPi use was particularly high) and IPO (where bevacizumab and PARPi use were similar).

For patients receiving more than 2L treatment, use of taxane monotherapy increased in all sites, except for Frankfurt. Use of trial compounds and ‘other’ chemotherapy also increased in most sites, as the proportion of patients receiving platinum combinations decreased (**Figure 6**). However, LTHT differs from other sites in its reduced use of trial compounds at 3L and notable use of platinum monotherapy at 3L (and 4L). Use of maintenance therapy after 3L decreased in all sites, except for Yonsei where 23.8% (50/210) of patients treated at 3L subsequently received bevacizumab maintenance.

### 3.5 Time to event analysis

Median OS in the overall cohort ranged from 25.5 months (IPO) to 65.5 months (Cluj) (**Table 4**). Apart from IPO, estimated median OS for patients with high-grade serous disease appeared to be similar at each site. Available data did not allow a statistical comparison of OS between sites. In the absence of systematic coded data on progression events, time between diagnosis and second line of SACT treatment initiation (time to 2L) was used as a proxy for disease progression. In the overall cohort, time to 2L ranged from 14.3 months (IPO) to 65.9 months (Yonsei). Median time to 2L was similar or shorter for patients with high grade serous disease, except for in IPO.

**Table 4:** Kaplan Meier estimates (in months) of time from diagnosis to the initiation of 2L SACT treatment (‘Time to 2L’) and of overall survival, by study site and showing estimates for whole cohort and patients with high-grade serous disease (‘HGS’).

	Cluj		Curie		Frankfurt		ICO		IPO		LTHT		Yonsei	
	median	95% CI	median	95% CI	median	95% CI	median	95% CI	median	95% CI	median	95% CI	median	95% CI
Time to 2L (all)	34.0	28.3 - 44.6	38.5	35.5 - 46.9	32.5	26.9 - 42.3	22.1	20.2 - 23.7	14.3	13.3 - 17.2	16.5	15.1 - 18	65.9	50.8 - *
Time to 2L (HGS)	27.0	23.2 - 30.7	30.2	26.5 - 36.1	29.4	23.3 - 35.4	20.8	19.5 - 23.2	17.7	14.9 - 22.4	15.4	14 - 16.7	30.8	25.2 - 37.7
Overall survival (all)	65.5	56.5 - 79.3	74.8	63.1 - *	59.0	49.3 - *	60.4	53.1 - *	25.5	19.6 - 35.4	30.1	27.6 - 35.3	*	
Overall survival (HGS)	63.0	26.7 – 33.2	74.3	66.1 - *	50.7	45.2 - *	58.4	53.1 – 75.3	50.8	34.0 - *	29.2	26.7 – 33.2	*	

*Overall survival estimates not reported because the estimated survival probability was above 50% or confidence interval could not be calculated.

## 4 Discussion

We describe a real-world outcomes study investigating treatment patterns of EOC in several European cancer centers and one from South Korea. This unfunded study was achieved using a hub and spoke analysis approach based on a common data model defined through clinical consensus between study sites. This approach ensured an efficient delivery of objectives across sites under different jurisdictions of GDPR as a first step to a full comparative outcomes study of treatment internationally.

Profiles of patient cohorts differed between study sites, with patients diagnosed in the Romanian (Cluj) and South Korean (Yonsei) centers being younger and tending to be diagnosed at an earlier stage of disease and with better ECOG performance score. Patients diagnosed in the UK center (LTHT) had the oldest age profile at diagnosis and this corresponded to advanced stage disease being diagnosed most frequently at this site, amongst patients with the poorest ECOG. Although the reported differences in patient profiles at diagnosis may in part reflect underlying population structure [it is notable that of all the study site countries, South Korea has the lowest proportion of women over 65 years of age in the general population (32)] or culture of health-seeking behavior, they might also indicate different national or even local health policy. It is notable that a significant proportion of the cohort studied would not usually be eligible for enrolment in clinical trials, meaning that real-world analysis of treatment patterns and outcomes may be of significant patient benefit.

Although the diverse use of debulking surgery as primary treatment for patients diagnosed at late stage appeared to be one such policy difference, examination of underlying clinical profiles suggests in fact a consistency of approach between study sites. The centers least likely to treat late-stage patients with debulking surgery also had the highest median age of overall patient cohort, and within the late-stage cohorts (regardless of study site) it tended to be older patients with poorer ECOG scores and higher rates of Stage IV disease that did not receive surgery. This presumably reflects a widespread selective use of surgery for those where success was most probable, the apparent differences between centers being explained by underlying patient profile rather than difference in policy.

Choice of platinum SACT at 1L was consistent across jurisdictions, reflecting an established evidence base for its use. The choice of platinum as monotherapy may be made where there is high risk of neurotoxicity following treatment (especially amongst older and frailer patients) (33, 34), and the particular use of monotherapy in LTHT is consistent with this cancer center having the highest proportion of patients (among the study sites) who were over 60 years old at diagnosis. Use of subsequent 1L maintenance therapy may be influenced by availability of drugs in each health system represented, efficacy of therapy for the patient cohort presenting and any side-effect profile that might steer clinician or patient choice. In Europe, the first PARPi, olaparib, was authorized in 2014 for 1L and 2L maintenance treatment (as monotherapy) of adult patients with relapsed, platinum−sensitive EOC (35) and its use for the study cohort in European centers, as reported here, is expected. However, olaparib was only approved in South Korea in 2019 (36) and this may explain its lack of reported use in this study.

Choice of 2L treatment has traditionally been directed by the PFI, the duration of response (up to progression) following completion of 1L platinum therapy and an indication of cancer sensitivity to platinum agents. Measures of PFI by center were not determined in this study, but the relatively high proportion of patients receiving 2L platinum at Frankfurt and Yonsei suggest higher proportions of patients with platinum-sensitive disease at these centers. Assuming similar timescales of diagnosis to treatment in each center, the increased median time to 2L reported here in Yonsei also support this observation. Hormone monotherapy was only used extensively in LTHT and IPO, as a 2L treatment option (and beyond). Studies have indicated improved outcomes using hormone therapy for patients with low-grade serous (LGS) disease (37), but the level of LGS is similar across centers in this study and use of hormone therapy may be a localized clinical preference (38). In LTHT, hormone therapy is often used for patients with low volume progressive disease (and reduced symptoms) to avoid more aggressive treatment with chemotherapy and potentially prolong the platinum free interval (SC; personal communication).

Beyond 2L, the inevitable development of resistance to platinum in patients with multiple recurrence means that PFI becomes a less significant marker for choice of therapy (27) and the decreasing use of platinum beyond 2L described here probably reflects this reduced sensitivity and response. Beyond trial-evidenced 2L treatments, patients are generally treated for ‘as long as possible’ to control disease (39), an approach perhaps evident here in all study sites, with some patients treated up to an eleventh line of SACT. In particular, a diversity of clinical trial study designs and sub-groups compared, and the lack of robust HRD testing in widespread use, has hampered the development of treatment algorithms for patients with non-BRCA mutated disease (14). There is little published evidence on the extent of treatment for recurrent disease and the high proportion of ‘trial/other chemotherapy’ reported here beyond 2L reflects the wide range of combinations and approaches tried in different centers (too many to reasonably categorize in our analysis). In this study, 978 patients were treated at 3L (32.1% of all those treated with SACT) and 563 at 4L (18.5%), showing the level of unmet treatment need for recurrent EOC in the real-world. Future analysis of this study cohort aims to better define the characteristics of this long-treated group of patients, with the hope that outcomes can be improved.

PARPi authorization in Europe was extended in 2018 for use as monotherapy following at least two lines of platinum treatment and in 2020 as maintenance in combination with bevacizumab (for patients with homologous recombination deficient (HRD)-positive advanced disease) (40). Data from the SOLO3 trial suggest that treatment of platinum-resistant BRCA-mutated disease with monotherapy PARPi is superior to non-platinum chemotherapy for those not previously receiving PARPi (41), but given the increasingly widespread use of PARPi as 1L maintenance, opportunities for its use in this way are limited (42). The study cohort reported mostly pre-dates these developments in the use of PARPi, and the use of these agents is restricted here to maintenance. The reason for the particularly strong adoption of PARPi maintenance in the German cancer center (in comparison to other European sites) is not known. A recent survey of treating clinicians in Germany found that side-effect profile and efficacy were the two main factors in their decision to use a PARPi (43) and the suspected high levels of platinum sensitivity in the German cohort (discussed above) may also be a factor. In two PARPi trials where quality of life indicators were included, no detrimental effect of PARPi maintenance therapy was shown (44).

This is the first report from an international collaboration established across 6 countries to investigate real-world treatment outcomes in EOC. The development of the CDM was a pragmatic approach to help establish the collaboration and demonstrate the potential to collect and compare high quality clinical data in EOC. Information on such a large real-world cohort (many of whom will not be represented in clinical trials) is crucial to deliver future patient benefit through optimized treatment approaches beyond 2L. The next stage of the partnership will seek to implement a more established CDM such as OMOP, developed through the Observational Health Data Sciences and Informatics (OHDSI) program, to facilitate the further expansion of the research network. A key limitation of the study is the information governance restriction on sharing and pooling patient-level data for analysis. To develop the analysis further, the collaboration is exploring the use of federated analytics to allow comparative outcome analysis.

## Data availability statement

The data analyzed in this study is subject to the following licenses/restrictions: Routine clinical data from multiple cancer centers. Data not pooled. Requests to access these datasets should be directed to geoff.hall2@nhs.net.

## Author contributions

All authors made substantial contributions to the conception or design of the work, but especially GH, MG-K, SC, WS, and BL. NN, AW, BL, HF, EM, MB, and PK acquired and analyzed data for the study. GH, SC, EJ, DK, SL, J-SF, FB, PK, PA-C, and CV provided interpretation of data for the work. WS, HF, and MG-K drafted the manuscript with critical revision contributed by all authors. All authors provided approval for publication of the content and agree to be accountable for all aspects of the work in relation to accuracy or integrity.
