# Supplementary material for: Ovarian Real-World International Consortium (ORWIC): A multicentre, real-world analysis of epithelial ovarian cancer treatment and outcomes
Source: Front Oncol. 2023 Jan 27;13:1114435. doi: 10.3389/fonc.2023.1114435 (PMC9911857; doi:10.3389/fonc.2023.1114435)
Supplement: Supplementary file 2 [file DataSheet_1.zip › openovary/html/00Index.html]

R: A package for analysing Ovarian Multi-site CDM data

# A package for analysing Ovarian Multi-site CDM data

---

## Documentation for package ‘openovary’ version 1.0

- DESCRIPTION file.

## Help Pages

|  |  |
| --- | --- |
| cdm\_labels | Categorical variable labels for common data model |
| check\_binary | Check binary variables |
| check\_date | Check date variables |
| check\_id | Check ID variables |
| check\_numeric | Check numeric variables |
| check\_var\_names | Check variable names |
| cols\_grad | Gradient colours |
| cols\_pack | A set of colours for plotting |
| cols\_preview | Preview the available colours |
| create\_risk\_table | Risk tables |
| export\_image | Export plot |
| label\_vars | Label categorical variables |
| long\_output | Write long output tables to file |
| plot\_cols | Plot colours |
| quantile\_df | Estimate quantiles for survival model |
| sample\_data\_import | A sample unlabelled dataset as would be imported. |
| sample\_data\_labelled | A sample labelled dataset for testing analysis functions. |
| strata\_cols | Strata colours |
| summarise\_var | Summarise variable |
| surv\_flatten | Flatten survival model |
| table\_values | Construct results table |
| tidy\_at\_risk | Tidy numbers at risk output tables |
| tidy\_survival | Tidy survival output tables |
| tidy\_table | Tidy output tables |
| tidy\_var\_names | Tidy up variable names |
| variable\_guide | Summary information for variables in the CDM |
