# Supplementary material for: Ovarian Real-World International Consortium (ORWIC): A multicentre, real-world analysis of epithelial ovarian cancer treatment and outcomes
Source: Front Oncol. 2023 Jan 27;13:1114435. doi: 10.3389/fonc.2023.1114435 (PMC9911857; doi:10.3389/fonc.2023.1114435)
Supplement: Supplementary file 2 [file DataSheet_1.zip › openovary/html/cdm_labels.html]

R: Categorical variable labels for common data model

|  |  |
| --- | --- |
| cdm\_labels {openovary} | R Documentation |

## Categorical variable labels for common data model

### Description

A dataset giving human readable labels for the numeric coding
of categorical variables in the CDM.
Does not include coding for binary and biomarker variables,
which are the same for each variable within each group.

### Usage

```
cdm_labels
```

### Format

A data frame with 68 rows and 3 variables:

variable
:   variable name, as given in the data guide

value
:   the numeric value of the variable level

label
:   the corresponding text label for the numeric value, for this variable

---

[Package *openovary* version 1.0 Index]
