# Supplementary material for: Ovarian Real-World International Consortium (ORWIC): A multicentre, real-world analysis of epithelial ovarian cancer treatment and outcomes
Source: Front Oncol. 2023 Jan 27;13:1114435. doi: 10.3389/fonc.2023.1114435 (PMC9911857; doi:10.3389/fonc.2023.1114435)
Supplement: Supplementary file 2 [file DataSheet_1.zip › openovary/html/check_date.html]

R: Check date variables

|  |  |
| --- | --- |
| check\_date {openovary} | R Documentation |

## Check date variables

### Description

Check that date variables are read in as the correct data format (string),
and have the correct date format (dd/mm/yyyy).

### Usage

```
check_date(data, date_var)
```

### Arguments

|  |  |
| --- | --- |
| `data` | data frame to check variables in. Required, no default. |
| `date_var` | name of date variable to check. Required, no default.  Warning messages are printed indicating missing, and unexpected variables. |

---

[Package *openovary* version 1.0 Index]
