# Supplementary material for: Ovarian Real-World International Consortium (ORWIC): A multicentre, real-world analysis of epithelial ovarian cancer treatment and outcomes
Source: Front Oncol. 2023 Jan 27;13:1114435. doi: 10.3389/fonc.2023.1114435 (PMC9911857; doi:10.3389/fonc.2023.1114435)
Supplement: Supplementary file 2 [file DataSheet_1.zip › openovary/html/check_id.html]

R: Check ID variables

|  |  |
| --- | --- |
| check\_id {openovary} | R Documentation |

## Check ID variables

### Description

Check ID variables meet requirements.

### Usage

```
check_id(data, var)
```

### Arguments

|  |  |
| --- | --- |
| `data` | data frame object containing variable. Required, no default. |
| `var` | variable name to check. Required, no default.  This function does not return an object. |

---

[Package *openovary* version 1.0 Index]
