# Supplementary material for: Ovarian Real-World International Consortium (ORWIC): A multicentre, real-world analysis of epithelial ovarian cancer treatment and outcomes
Source: Front Oncol. 2023 Jan 27;13:1114435. doi: 10.3389/fonc.2023.1114435 (PMC9911857; doi:10.3389/fonc.2023.1114435)
Supplement: Supplementary file 2 [file DataSheet_1.zip › openovary/html/check_numeric.html]

R: Check numeric variables

|  |  |
| --- | --- |
| check\_numeric {openovary} | R Documentation |

## Check numeric variables

### Description

Check numeric variables are valid and print summaries of data rule violations.

### Usage

```
check_numeric(
  data,
  var,
  maxval,
  minval = 0,
  discrete = F,
  digits = NULL,
  missingval = NULL,
  compare_vector = NULL,
  direction = "smaller"
)
```

### Arguments

|  |  |
| --- | --- |
| `data` | data frame holding variable to check. Required. |
| `var` | name of variable to check. Required. |
| `maxval` | maximum valid value for the variable. Required, no default. |
| `minval` | minimum valid value for the variable. Required, default is 0. |
| `discrete` | whether data is continuous (FALSE) or discrete (TRUE). Required, default is FALSE. |
| `digits` | how many digits valid values should have. E.g. for a year in YYYY format, this would be 4. Optional, no default. |
| `missingval` | a value indicating valid missing unknown data other than NA. Optional, no default. |
| `compare_vector` | the name of a vector to compare a numeric vector to. For example, setting as "surv\_time" would compare values in var to survival time. Optional, no default. |
| `direction` | whether the values in var should be "smaller" (default) or not.  No data objects are returned from the function. NA's are removed before checking the data, but the original data supplied is not overwritten. |

---

[Package *openovary* version 1.0 Index]
