# Supplementary material for: Ovarian Real-World International Consortium (ORWIC): A multicentre, real-world analysis of epithelial ovarian cancer treatment and outcomes
Source: Front Oncol. 2023 Jan 27;13:1114435. doi: 10.3389/fonc.2023.1114435 (PMC9911857; doi:10.3389/fonc.2023.1114435)
Supplement: Supplementary file 2 [file DataSheet_1.zip › openovary/html/check_var_names.html]

R: Check variable names

|  |  |
| --- | --- |
| check\_var\_names {openovary} | R Documentation |

## Check variable names

### Description

Check that the variable names (column headers) match those expected.

### Usage

```
check_var_names(data, expected_vars)
```

### Arguments

|  |  |
| --- | --- |
| `data` | data frame to check variable names in. Required, no default. |
| `expected_vars` | vector of expected variable names. Required, no default.  Warning messages are printed indicating missing, and unexpected variables. |

### Value

Returns a list of length 2.
$missing\_vars lists the variables missing from those expected.
$extra\_vars lists variables in the data that are not expected for the analysis.

---

[Package *openovary* version 1.0 Index]
