# Supplementary material for: Ovarian Real-World International Consortium (ORWIC): A multicentre, real-world analysis of epithelial ovarian cancer treatment and outcomes
Source: Front Oncol. 2023 Jan 27;13:1114435. doi: 10.3389/fonc.2023.1114435 (PMC9911857; doi:10.3389/fonc.2023.1114435)
Supplement: Supplementary file 2 [file DataSheet_1.zip › openovary/html/cols_grad.html]

R: Gradient colours

|  |  |
| --- | --- |
| cols\_grad {openovary} | R Documentation |

## Gradient colours

### Description

Generate a gradient of colours between specified colours

### Usage

```
cols_grad(..., n = NULL, rgb = FALSE)
```

### Arguments

|  |  |
| --- | --- |
| `...` | names of colours from the standard palette. Can be any length but generally should be fewer than the number of steps requested in n. Available colours are: blue, darkblue, green, darkgreen, teal, ochre, orange, purple, red and gray. |
| `n` | number of steps for the gradient to have between the provided colours. Required, no default. Must be at least 2, not fewer than the number of colours provided. Can be the same as the number of colours provided, but this will produce a warning that this can't really generate a gradient. |
| `rgb` | TRUE FALSE whether to return the colours as RGB vectors (TRUE) or not (FALSE). Optional, default is FALSE. |

### Value

Returns a vector of length n, with colour values specified at n-1
intervals between the first and last colour provided.
Through the other colours provided (if any).

---

[Package *openovary* version 1.0 Index]
