# Supplementary material for: Ovarian Real-World International Consortium (ORWIC): A multicentre, real-world analysis of epithelial ovarian cancer treatment and outcomes
Source: Front Oncol. 2023 Jan 27;13:1114435. doi: 10.3389/fonc.2023.1114435 (PMC9911857; doi:10.3389/fonc.2023.1114435)
Supplement: Supplementary file 2 [file DataSheet_1.zip › openovary/html/cols_pack.html]

R: A set of colours for plotting

|  |  |
| --- | --- |
| cols\_pack {openovary} | R Documentation |

## A set of colours for plotting

### Description

A list object of length 2, with reference codes for 10
colours for use in plotting.

### Usage

```
cols_pack
```

### Format

A list of length 2:

colours
:   a vector of length 10, giving the hex codes for each colour

RGB\_colours
:   a list of length 10, containing vectors of length 3
    giving RGB codes for each colour

---

[Package *openovary* version 1.0 Index]
