# Supplementary material for: Ovarian Real-World International Consortium (ORWIC): A multicentre, real-world analysis of epithelial ovarian cancer treatment and outcomes
Source: Front Oncol. 2023 Jan 27;13:1114435. doi: 10.3389/fonc.2023.1114435 (PMC9911857; doi:10.3389/fonc.2023.1114435)
Supplement: Supplementary file 2 [file DataSheet_1.zip › openovary/html/cols_preview.html]

R: Preview the available colours

|  |  |
| --- | --- |
| cols\_preview {openovary} | R Documentation |

## Preview the available colours

### Description

The function has no arguments.
Generates a simple scatter plot to preview the available colours.
Showing the names of the colours.

### Usage

```
cols_preview()
```

### Value

Returns a scatter plot with a point for each colour in the palette,
and their corresponding names.

---

[Package *openovary* version 1.0 Index]
