# Supplementary material for: Ovarian Real-World International Consortium (ORWIC): A multicentre, real-world analysis of epithelial ovarian cancer treatment and outcomes
Source: Front Oncol. 2023 Jan 27;13:1114435. doi: 10.3389/fonc.2023.1114435 (PMC9911857; doi:10.3389/fonc.2023.1114435)
Supplement: Supplementary file 2 [file DataSheet_1.zip › openovary/html/create_risk_table.html]

R: Risk tables

|  |  |
| --- | --- |
| create\_risk\_table {openovary} | R Documentation |

## Risk tables

### Description

create\_risk\_table is a wrapper function for ggsurvtable
That can accept non evenly spaced timepoints
And produces a tidier risk table suitable to write to a .csv file.

### Usage

```
create_risk_table(fit = NULL, timepoints = c(1, 2, 5, 10))
```

### Arguments

|  |  |
| --- | --- |
| `fit` | Survival model to generate risk table for. Required, no default. |
| `timepoints` | Vector of time points (years) at which to summarise survival data. Required, default is 1, 2, 5 and 10 years. |

### Value

Returns a risk table summarising survival status at the points in timepoints.

---

[Package *openovary* version 1.0 Index]
