# Supplementary material for: Ovarian Real-World International Consortium (ORWIC): A multicentre, real-world analysis of epithelial ovarian cancer treatment and outcomes
Source: Front Oncol. 2023 Jan 27;13:1114435. doi: 10.3389/fonc.2023.1114435 (PMC9911857; doi:10.3389/fonc.2023.1114435)
Supplement: Supplementary file 2 [file DataSheet_1.zip › openovary/html/export_image.html]

R: Export plot

|  |  |
| --- | --- |
| export\_image {openovary} | R Documentation |

## Export plot

### Description

Export a plot object as an image

### Usage

```
export_image(plotobj, filename)
```

### Arguments

|  |  |
| --- | --- |
| `plotobj` | plottable object to save to file. Required, no default. |
| `filename` | name for the exported file, required. Default is current system time. |

### Value

Returns a plot object in the current directory, as a 300dpi landscape A4 png file

---

[Package *openovary* version 1.0 Index]
