# Supplementary material for: Ovarian Real-World International Consortium (ORWIC): A multicentre, real-world analysis of epithelial ovarian cancer treatment and outcomes
Source: Front Oncol. 2023 Jan 27;13:1114435. doi: 10.3389/fonc.2023.1114435 (PMC9911857; doi:10.3389/fonc.2023.1114435)
Supplement: Supplementary file 2 [file DataSheet_1.zip › openovary/html/label_vars.html]

R: Label categorical variables

|  |  |
| --- | --- |
| label\_vars {openovary} | R Documentation |

## Label categorical variables

### Description

Labels variables using either a lookup table, or pre set values.
The default values for lookupvarcol, lookupvalcol, and lookuplabelcol
can be used to use the lookup file "cdm\_labels" provided with the package.

### Usage

```
label_vars(
  data,
  var,
  type = "binary",
  lookup = NULL,
  lookupvarcol = "variable",
  lookupvalcol = "value",
  lookuplabelcol = "label"
)
```

### Arguments

|  |  |
| --- | --- |
| `data` | a data frame, required. |
| `var` | the name of a variable in the data frame specified in data, required. |
| `type` | the data type of the variable in var, required. Default is "binary". Can be "binary" (yes no), "biomarker" ("Wildtype", "Pathogenic Variant", "Variant of unknown significance") or "coded". |
| `lookup` | a lookup file for variable labelling. Optional. Required if type is "coded". A data frame with a column listing the variable name, expected values, and their corresponding labels. |
| `lookupvarcol` | The name of the column in the lookup data frame giving the variable name. Default: "variable". |
| `lookupvalcol` | The name of the column in the lookup data frame giving the variable values. Default: "value" |
| `lookuplabelcol` | The name of the column in the lookup data frame giving the value labels. Default: label. |

### Value

a vector of the same length as that provided in "var",
of class "factor".
If type is "binary" (default), the vector has the levels 0,1, labelled as "Yes","No".
If type is "biomarker", the vector has levels 0,1,2, labelled as
"Wildtype", "Pathogenic Variant", "Variant of unknown significance".
If type is "coded numeric", the vector has levels and labels as specified in the lookup.

NOTE: values in the vector that are not in the specified levels, will replace with the same level,
labelled as "Missing Invalid."
A warning message will print indicating the number of affected rows.

### Examples

```
tidy_var_names( patient_data )
```

---

[Package *openovary* version 1.0 Index]
