# Supplementary material for: Ovarian Real-World International Consortium (ORWIC): A multicentre, real-world analysis of epithelial ovarian cancer treatment and outcomes
Source: Front Oncol. 2023 Jan 27;13:1114435. doi: 10.3389/fonc.2023.1114435 (PMC9911857; doi:10.3389/fonc.2023.1114435)
Supplement: Supplementary file 2 [file DataSheet_1.zip › openovary/html/long_output.html]

R: Write long output tables to file

|  |  |
| --- | --- |
| long\_output {openovary} | R Documentation |

## Write long output tables to file

### Description

Writes long form data for multi site models to csv.

### Usage

```
long_output(result, filename = NULL, output_tag = NULL)
```

### Arguments

|  |  |
| --- | --- |
| `table_in` | Table produced by table\_values. Required, no default. |
| `output_label` | Additional label to add to file name. Optional, default uses the name of the object provided to "result". |

### Value

Does not return an object, writes the table out to a .csv.

---

[Package *openovary* version 1.0 Index]
