# Supplementary material for: Ovarian Real-World International Consortium (ORWIC): A multicentre, real-world analysis of epithelial ovarian cancer treatment and outcomes
Source: Front Oncol. 2023 Jan 27;13:1114435. doi: 10.3389/fonc.2023.1114435 (PMC9911857; doi:10.3389/fonc.2023.1114435)
Supplement: Supplementary file 2 [file DataSheet_1.zip › openovary/html/plot_cols.html]

R: Plot colours

|  |  |
| --- | --- |
| plot\_cols {openovary} | R Documentation |

## Plot colours

### Description

Generate colours for a plot from a standard palette

### Usage

```
plot_cols(..., rgb = FALSE, colvec = NULL)
```

### Arguments

|  |  |
| --- | --- |
| `...` | names of colours to obtain as character strings. No default. Required if colvec==NULL Available colours are: blue, darkblue, green, darkgreen, teal, ochre, orange, purple, red and gray. |
| `rgb` | TRUE FALSE. Whether to return colours as vectors of length 3 giving RGB values. Optional, default is NULL. |
| `colvec` | colours provided as a named vector. Mostly used when this function is used internally by other functions, e.g. cols\_grad. Optional, no default. |

### Value

Returns a vector of the same length as the number of colours requested,
containing hex references for the colours requested from the standard palette.

---

[Package *openovary* version 1.0 Index]
