# Supplementary material for: Ovarian Real-World International Consortium (ORWIC): A multicentre, real-world analysis of epithelial ovarian cancer treatment and outcomes
Source: Front Oncol. 2023 Jan 27;13:1114435. doi: 10.3389/fonc.2023.1114435 (PMC9911857; doi:10.3389/fonc.2023.1114435)
Supplement: Supplementary file 2 [file DataSheet_1.zip › openovary/html/quantile_df.html]

R: Estimate quantiles for survival model

|  |  |
| --- | --- |
| quantile\_df {openovary} | R Documentation |

## Estimate quantiles for survival model

### Description

Estimate quantiles for a survival model, and produce tidy output.

### Usage

```
quantile_df(fit, prob)
```

### Arguments

|  |  |
| --- | --- |
| `fit` | required, no default. The fitted model to summarise. ' |
| `prob` | the quantile to estimate time to.= |

### Value

Returns a
data frame with the estimated quantile survival overall, and in each strata group of the
model, along with corresponding confidence intervals. '

---

[Package *openovary* version 1.0 Index]
