# Supplementary material for: Ovarian Real-World International Consortium (ORWIC): A multicentre, real-world analysis of epithelial ovarian cancer treatment and outcomes
Source: Front Oncol. 2023 Jan 27;13:1114435. doi: 10.3389/fonc.2023.1114435 (PMC9911857; doi:10.3389/fonc.2023.1114435)
Supplement: Supplementary file 2 [file DataSheet_1.zip › openovary/html/sample_data_import.html]

R: A sample unlabelled dataset as would be imported.

|  |  |
| --- | --- |
| sample\_data\_import {openovary} | R Documentation |

## A sample unlabelled dataset as would be imported.

### Description

A list object of length 3, each containing a data frame
that is a sample of one of the 3 data tables as described
in the data guide.

### Usage

```
sample_data_import
```

### Format

A list of length 3:

patient
:   a sample patient table, 100 patient rows and 38 variables

surgery
:   a sample surgery events table, 100 surgery event rows and 6 variables

sact
:   a sample sact events table, 100 sact event rows and 7 variables

---

[Package *openovary* version 1.0 Index]
