# Supplementary material for: Ovarian Real-World International Consortium (ORWIC): A multicentre, real-world analysis of epithelial ovarian cancer treatment and outcomes
Source: Front Oncol. 2023 Jan 27;13:1114435. doi: 10.3389/fonc.2023.1114435 (PMC9911857; doi:10.3389/fonc.2023.1114435)
Supplement: Supplementary file 2 [file DataSheet_1.zip › openovary/html/strata_cols.html]

R: Strata colours

|  |  |
| --- | --- |
| strata\_cols {openovary} | R Documentation |

## Strata colours

### Description

Generate a gradient of colours for plotting survival fits

### Usage

```
strata_cols(
  fit,
  col = "gray",
  col2 = "blue",
  legend.labs = NULL,
  gradient = TRUE,
  col_vec = NULL
)
```

### Arguments

|  |  |
| --- | --- |
| `fit` | A survival model fit. Required, no default. |
| `col` | Starting colour. Required, default is gray. |
| `col2` | End colour. Required, default is blue. |
| `legend.labs` | a vector of names for the strata levels the colours are to be assigned to. Optional, no default. |
| `gradient` | TRUE/FALSE, whether the function should produce a gradient of colours (TRUE), or use a provided vector (FALSE). Optional, default is FALSE. |
| `col_vec` | Vector of colours. Optional, no default. Required if gradient=FALSE. |

### Value

Returns a vector of colours, of the same length as the number
of strata levels in the fit object.

---

[Package *openovary* version 1.0 Index]
