# Supplementary material for: Ovarian Real-World International Consortium (ORWIC): A multicentre, real-world analysis of epithelial ovarian cancer treatment and outcomes
Source: Front Oncol. 2023 Jan 27;13:1114435. doi: 10.3389/fonc.2023.1114435 (PMC9911857; doi:10.3389/fonc.2023.1114435)
Supplement: Supplementary file 2 [file DataSheet_1.zip › openovary/html/summarise_var.html]

R: Summarise variable

|  |  |
| --- | --- |
| summarise\_var {openovary} | R Documentation |

## Summarise variable

### Description

For continuous variables, reports: mean, SD, median, IQR, total values, and total missing.
For discrete, reports: count and percentage in each group, including missing values.

### Usage

```
summarise_var(vname, data, continuous_var = FALSE)
```

### Arguments

|  |  |
| --- | --- |
| `vname` | name of variable to summarise. Vector of length 1. Required, no default. |
| `data` | data frame containing var |
| `continuous_var` | "TRUE" or "FALSE" whether variable is continuous numeric or not. Required, default is "FALSE." |

### Value

Returns a data frame with a row for each result,
and a column for the variable, the variable level (where appropriate),
the result stated, and the value of the result for this group in the variable.

---

[Package *openovary* version 1.0 Index]
