# Supplementary material for: Ovarian Real-World International Consortium (ORWIC): A multicentre, real-world analysis of epithelial ovarian cancer treatment and outcomes
Source: Front Oncol. 2023 Jan 27;13:1114435. doi: 10.3389/fonc.2023.1114435 (PMC9911857; doi:10.3389/fonc.2023.1114435)
Supplement: Supplementary file 2 [file DataSheet_1.zip › openovary/html/surv_flatten.html]

R: Flatten survival model

|  |  |
| --- | --- |
| surv\_flatten {openovary} | R Documentation |

## Flatten survival model

### Description

Flatten (summarise) results from a survival model fit, to export to a table.

### Usage

```
surv_flatten(
  fit,
  site,
  outcome_label = "Overall Survival",
  include_quartiles = TRUE
)
```

### Arguments

|  |  |
| --- | --- |
| `fit` | required, no default. The fitted model to summarise. ' |
| `site` | the name of the site the results are from. |
| `outcome_label` | optional, default "Overall survival". Label for results. |

### Value

Returns a
data frame with the median survival overall, and in each strata group of the
model, along with corresponding confidence intervals. '

---

[Package *openovary* version 1.0 Index]
