# Supplementary material for: Ovarian Real-World International Consortium (ORWIC): A multicentre, real-world analysis of epithelial ovarian cancer treatment and outcomes
Source: Front Oncol. 2023 Jan 27;13:1114435. doi: 10.3389/fonc.2023.1114435 (PMC9911857; doi:10.3389/fonc.2023.1114435)
Supplement: Supplementary file 2 [file DataSheet_1.zip › openovary/html/table_values.html]

R: Construct results table

|  |  |
| --- | --- |
| table\_values {openovary} | R Documentation |

## Construct results table

### Description

Construct table of variable summaries, generated as described in the SAP.
For continuous variables, reports: mean, SD, median, IQR, total values, and total missing.
For discrete, reports: count and percentage in each group, including missing values.

### Usage

```
table_values(
  varnames,
  data,
  continuous_vars = NULL,
  subpopulation = NULL,
  include_total = TRUE
)
```

### Arguments

|  |  |
| --- | --- |
| `varnames` | vector of variable names in data to summarise. Required, no default. |
| `data` | data frame containing study data to summarise. Required, no default. |
| `continuous_vars` | vector of the same length or less than varnames, a subset of varnames specifying the variables that are continuous numeric data. Optional, with no default. |
| `subpopulation` | name of a variable in the object data, to be used as a sub population. i.e. the results are generated for each sub group of the variable specified. Optional, no default. |
| `include_total` | "TRUE" or "FALSE", whether to include summaries for the whole cohort in data. Required, default is "TRUE." Must be "TRUE" if a sub population is not specified, or no output can be generated. |

### Value

Returns a data frame with a column each for the variable being
summarised, and (where relevant) the level of that variable, the result
(i.e. count, percentage, mean, standard deviation etc.), and the value for
this result for that group. Where a subpopulation is provided, two addition
columns give the sub population, and the level (group) in the sub
population the results refer to. The data frame has one row per result, per
group.

---

[Package *openovary* version 1.0 Index]
