# Supplementary material for: Ovarian Real-World International Consortium (ORWIC): A multicentre, real-world analysis of epithelial ovarian cancer treatment and outcomes
Source: Front Oncol. 2023 Jan 27;13:1114435. doi: 10.3389/fonc.2023.1114435 (PMC9911857; doi:10.3389/fonc.2023.1114435)
Supplement: Supplementary file 2 [file DataSheet_1.zip › openovary/html/tidy_table.html]

R: Tidy output tables

|  |  |
| --- | --- |
| tidy\_table {openovary} | R Documentation |

## Tidy output tables

### Description

Converts long form data for multi site models to single site tabes

### Usage

```
tidy_table(
  result,
  write_csv = TRUE,
  filename = NULL,
  output_tag = NULL,
  keep_table = TRUE
)
```

### Arguments

|  |  |
| --- | --- |
| `result` | Table produced by table\_values. |
| `write_csv` | TRUE/FALSE, when TRUE will write the resulting table to a .csv file. Optional, default is TRUE. |
| `filename` | filename for writing out to .csv. Needed if write\_csv==TRUE. Default is oenovaR filename set at top of script (filename). |
| `keep_table` | TRUE/FALSE, when TRUE will save the table to an R object. Default is TRUE. |
| `output_tab` | a tag for the output file. Optional, default is the name of the object provided to result. |

### Value

A formatted table that can be written out to e.g. Excel.

---

[Package *openovary* version 1.0 Index]
