# Supplementary material for: Ovarian Real-World International Consortium (ORWIC): A multicentre, real-world analysis of epithelial ovarian cancer treatment and outcomes
Source: Front Oncol. 2023 Jan 27;13:1114435. doi: 10.3389/fonc.2023.1114435 (PMC9911857; doi:10.3389/fonc.2023.1114435)
Supplement: Supplementary file 2 [file DataSheet_1.zip › openovary/html/tidy_var_names.html]

R: Tidy up variable names

|  |  |
| --- | --- |
| tidy\_var\_names {openovary} | R Documentation |

## Tidy up variable names

### Description

Tidies variable (column header) names from input data file,
to make handling in analysis code easier.

### Usage

```
tidy_var_names(data)
```

### Arguments

|  |  |
| --- | --- |
| `data` | a data frame |

### Value

the data frame provided as data
with variable names all in lower case, and all punctuation replaced
with underscores (\_).

### Examples

```
tidy_var_names( patient_data )
```

---

[Package *openovary* version 1.0 Index]
