# Supplementary material for: Ovarian Real-World International Consortium (ORWIC): A multicentre, real-world analysis of epithelial ovarian cancer treatment and outcomes
Source: Front Oncol. 2023 Jan 27;13:1114435. doi: 10.3389/fonc.2023.1114435 (PMC9911857; doi:10.3389/fonc.2023.1114435)
Supplement: Supplementary file 2 [file DataSheet_1.zip › openovary/html/variable_guide.html]

R: Summary information for variables in the CDM

|  |  |
| --- | --- |
| variable\_guide {openovary} | R Documentation |

## Summary information for variables in the CDM

### Description

A dataset giving some summary details of the variables in the CDM.

### Usage

```
variable_guide
```

### Format

A data frame with 62 rows and 4 variables:

table
:   the table where the variable is located

name
:   the variable name

interim
:   yes/no, whether the variable is needed for the interim results

type
:   for categorical variables, the type. One of coded, binary, or biomarker

---

[Package *openovary* version 1.0 Index]
