# Supplementary material for: Ovarian Real-World International Consortium (ORWIC): A multicentre, real-world analysis of epithelial ovarian cancer treatment and outcomes
Source: Front Oncol. 2023 Jan 27;13:1114435. doi: 10.3389/fonc.2023.1114435 (PMC9911857; doi:10.3389/fonc.2023.1114435)
Supplement: Supplementary file 2 [file DataSheet_1.zip › openovary/manual_openovary.pdf]

openovary: an R Package to enable aligned analysis of real worl international multi-site ovarian cancer data

Beth Levick & the ORWIC team

2022-03-18

Manual

Introduction

The oenovaR package is a stand-alone package that is intended **solely** for use analysing data set up using the CDM in Levick *et. al*. **The functions and code should not be used for any other purpose**.

Getting started

Recommended set up

All the analysis scripts are provided in the R Statistical Programming Language. Version 3.6.0 ("Planting of a tree") or later should be installed.

It is highly recommended to have RStudio (version 1.1 or later) installed. This allows for the R Markdown helper files to be used, described in the section below.

It is also recommended to have Microsoft Word installed.

R and RStudio are available for Windows, Macintosh and Linux operating systems.

Dependencies

The package uses several other R packages available on CRAN. These should be installed as you install openovary in R.

If this fails, these can be installed from your preferred CRAN repository, to wherever you normally save R packages by running the following command in the R Console:

```
install.packages(c("survival", "survminer", "data.table", "dplyr", "ggplot2",
"knitr"))
```

If the analysis data is not stored as a .csv file, the packages "haven" and "xlsx" provide methods for reading in SAS, STATA and Excel files.

Installing oenovaR

oenovaR is not available on CRAN, and can only be downloaded from the project Git repository. To install the functions:

1. Download or pull the latest release of the compressed (.zip) file from the Git, and save to your preferred file directory.
2. In RStudio, go to the "Tools" menu and select "install packages…"

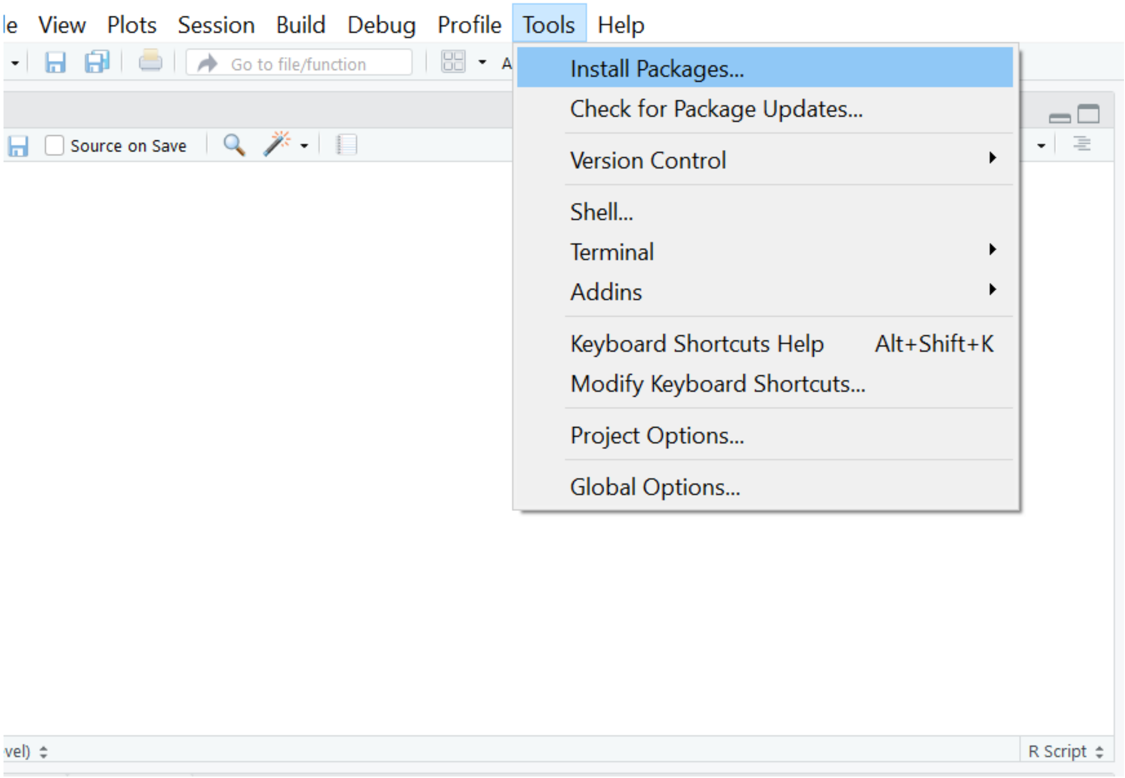

Figure 1. Manually install packages.

3. Select "install from compressed file" in the location drop down box, and then use the browse button to locate the oenovaR.zip file.

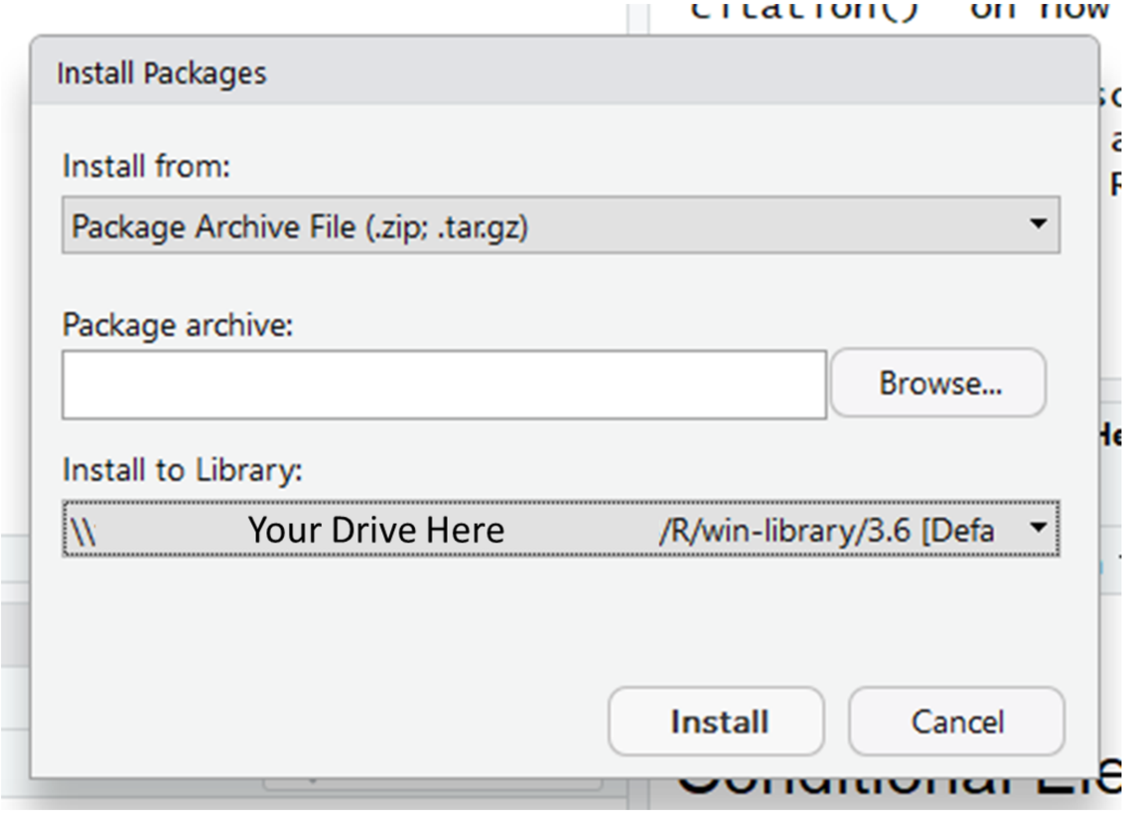

Figure 2. Install compressed package file.

4. Select install to install the package to your normal R Library.

If you have a previous verison installed, uninstall this using remove.packages("oenovaR") in R, and then repeating the steps above to reinstall.

Using the script files

Locating the script files

Two scripts will be provided for each stage of the analysis (interim and final analysis), a validation script and an analysis script. These will be available on the OEN Ovarian Sharepoint sites, and downloaded with the oenovaR package.

After the package has downloaded, navigate to the "oenovaR" folder in your R library file directory. Inside the "oenovaR" file will be four files:

- "validation.Rmd" and "analysis.Rmd" RMarkdown files to run the interim analysis.
- "interim_validation.R" and "interim_analysis.R" the accompanying simple R files, if it is not possible to use the RMarkdown files.

Editing site specific variables

In each file, there are some variables that need to be edited by each site. This may be just to put the site name into a variable, or to provide the proper file paths for data files. Wherever this is required, comments in the script should indicate what changes are required.

R Markdown files

The recommended method to run the analysis is to use the R Markdown (.Rmd) files, in the "oenovaR" folder with ".Rmd" file extensions. These can be opened in RStudio.

R Markdown files contain chunks of R code to run the analysis and information in comments, like a normal R script. They also contain sections of code that will print the results to a document (in this case a Microsoft Word .docx file), to quickly produce formatted reports.

More information on R Markdown Files is available on the RStudio website.

Alternatives to RMarkdown

Versions of the R Script are available as .R files, without the markdown functionality (files in the "oenovaR" directory with ".R" extensions). These contain scripts to conduct the same analysis, but that will not automatically produce associated reports.

These files should only be used where it is not possible to use the markdown files (e.g. if it is not possible to install R Studio). In this case, it is recommend to have another IDE such as Notepad++ to inspect and edit the files as appropriate, before running sections or whole scripts in R.

If you will not be working with the RMarkdown files, please inform Beth Levick (bethany.levick@nhs.net).

RMarkdown workflow

The recommended workflow for using each scripts is:

1. Edit any site specific information as described above.

2. Run each code chunk in turn, using the button in the script.

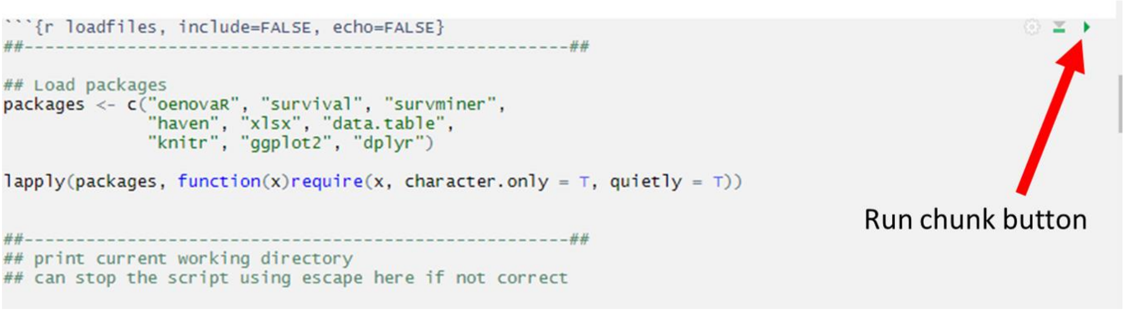

Figure 3. "Play" button to run individual code chunk.

3. Once all the chunks can run successfully, "knit" the document using the "knit" button in RStudio.

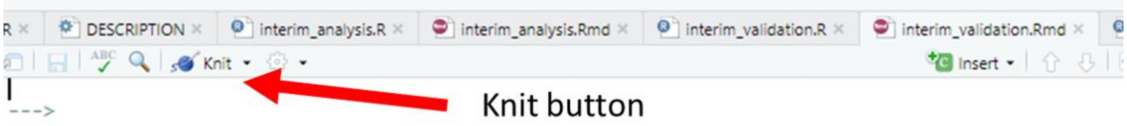

Figure 3. "Knit" button to run entire script and produce automated report.

Script actions

For each stage of the analysis (interim and final) there will be two scripts, a validation script and an analysis script. These scripts use the functions in the oenovaR package to run the analysis as described in the SAP.

The validation script

The validation script file should be run first. This will check two main things:

- Which variables are available, which are missing, and if any are provided that are not expected. In short, this compares the column headers of the provided data to those expected in the CDM.
- Whether each variable's content is as expected, as described in the data guide (i.e. coding, valid minimum and maximum values).

The RMarkdown file will produce a report as a Word Document detailing any differences between the provided data and the expected CDM. This should be sent to the Leeds team as soon as possible, to confirm if the analysis data is appropriate for use in the analysis.

If there are sufficient problems with the analysis data, it may be requested that this is updated before any analytical results can be accepted. If the differences are minor, or cannot be rectified, this will be noted when the multisite results are reported.

The analysis script

The analysis script will then use the approved CDM analysis data to produce the statistical output described in the SAP. Each step is described further in the script comments.

The previous knitted analysis results document is now not produced. Output files are created as follows:

1. Tables are output in long form (for the multi-site analysis), and a more readable ‘tidy’ form. These are saved as .csv files.

2. A .png file for each figure.

The long form .csv files contain all the results needed for the multi site comparisons, and these are the information required to be sent to the Leeds site.

These tables will be compiled to produce a Shiny R dashboard, allowing for interactive investigation of the results.
